# Lab-in-a-Fiber detection and capture of cells

**DOI:** 10.1038/s41598-025-92585-6

**Published:** 2025-03-20

**Authors:** João C. Varela, Achar V. Harish, Pawel Maniewski, Timothy Gibbon, Oana Tudoran, Rainer Heuchel, Matthias Löhr, Walter Margulis, Aman Russom, Fredrik Laurell

**Affiliations:** 1 Division of Nanobiotechnology, Department of Protein Science, Science for Life Laboratory, KTH Royal Institute of Technology, Solna, Sweden; 2 AIMES Center for the Advancement of Integrated Medical and Engineering Sciences, Karolinska Institutet and KTH Royal Institute of Technology, Stockholm, Sweden; 3 Department of Applied Physics, KTH Royal Institute of Technology, Stockholm, Sweden; 4 Research Institutes of Sweden (RISE), Stockholm, Sweden; 5 Department of Genetics, Genomics and Experimental Pathology, The Oncology Institute “Prof. Dr. Ion Chiricuta”, Cluj-Napoca, Romania; 6 Department of Clinical Science, Intervention and Technology, Karolinska Institutet, Stockholm, Sweden; 7 Department of Cancer Medicine, Division for Upper GI, Karolinska University Hospital, Stockholm, Sweden; 8 Catholic University of Rio de Janeiro PUC-Rio, Rio de Janeiro, Brazil

**Keywords:** Lab-in-a-Fiber, Cell detection, Cell capture, Cancer diagnostics, Cancer, Optics and photonics, Physics

## Abstract

A lab-in-a-fiber component was fabricated using an optical fiber and a fiber capillary. It was used in a test suspension of fluorescently labeled and unlabeled cells and enabled detection of the labeled cells. Subsequently the labeled cells were selectively collected via suction into the capillary. A novel sampling technique reduced photobleaching of the labeled cells, extending the measurement time. The collected cells remained viable for downstream analysis. This platform’s low fabrication cost, simplicity, compatibility with standard laboratory equipment, and capacity for fully automated cell capture highlights its potential for future applications in minimally invasive sample collection and point-of-care diagnostics. We demonstrate this LiF device to showcase the capability of optical fiber technology in creating low-cost, low-complexity cancer diagnostic devices. Furthermore, the LiF device holds promise for in vivo diagnostics, facilitating cell isolation and analysis.

**Supplementary Information:**

The online version contains supplementary material available at 10.1038/s41598-025-92585-6.

## Introduction

Isolation of individual single cells or small clusters is essential for analyzing heterogeneous cell populations. Identifying and isolating relevant subpopulations is crucial for understanding these complex systems while also helping spot rare cell types that could drive disease progression, which is usually overlooked by traditional bulk analysis methods. Detecting, isolating, and analyzing these cells would thus present valuable opportunities for early disease detection, therapeutic intervention, and treatment monitoring^1^. Furthermore, the isolation of single cells or small clusters also facilitates various downstream analysis methods^2^, enabling a comprehensive profiling of the collected samples for monitoring disease progression and treatment efficacy.

In cancer diagnostics, targeted tissue biopsy remains the gold standard for the detection, acquisition, and analysis of tumoral cells^3^. Fine needle aspiration is one of the preferred diagnostic tools for investigating tumors, being a simple, cost-effective, and easy to perform method that offers an accuracy similar to other biopsy techniques^4,5^. While this and other biopsy procedures generally pose minimal complications for patients with superficial tumors, deeper and harder-to-reach cancers (e.g.: pancreatic and lung cancer) often require more invasive procedures, which carry higher risks^5^. At the same time, repeated biopsies have been associated with further complications for the patient^6,7^, preventing its routine use for more frequent screenings. To circumvent this, liquid biopsies have emerged as a less invasive option for cancer diagnosis, collecting body fluids containing secreted extracellular vesicles, proteins, or other biomarkers that can be used to assess the tumor^8–10^. Despite its usefulness, liquid biopsy also presents some obstacles, namely in the detection of very low concentrations of target molecules that vary significantly depending on the sample of choice. As such, these technologies are often used as complementary clinical information, thus not completely avoiding the need for more invasive biopsies^10–12^. Hence, there is a need for improved diagnostics technologies both for in-vitro and in-vivo scenarios.

Lab-on-Fiber or Lab-in-a-Fiber (LiF) technologies emerge as compact and versatile analytical platforms, whose small dimensions, flexibility, and chemical inertness present ideal characteristics for less invasive, in-vivo and in-vitro point-of-care diagnostics^13–15^. By integrating modern nanotechnologies into typically inert, light-coupled platforms, it is possible to create miniaturized systems capable of performing complex biosensing assays, while retaining unparalleled performance levels^13,14,16^. For example, a biconical tapered fiber sensor has been used to detect the pathogen E. Coli^17^. In another work, a Ω-shaped fiber sensor was used for detecting cell-free DNA^18^. Ref. SERS with fiber tip has also been described for detection of cancer^19^. These technologies are capable of detecting various cancer biomarkers with improved sensitivities^20,21^, while their small size has allowed the screening of the gastrointestinal tract with minimal invasiveness^22,23^. LiF platforms can also be combined with Artificial Intelligence (AI) to perform improved disease screening and diagnosis^15,24,25^.

Here, we build on our group’s previous developments^26,27^ to create an all-fiber, low-cost LiF device capable of detecting and capturing individual cells or small clusters. In this work, we combine the light-guiding of optical fibers with capillaries aimed at fluid handling. The fiber device was scanned through the solution and the fluorescence signal was collected and analyzed so fluorescent cells in the vicinity of the fiber tip could be captured. The use of this cell collection system can be envisaged in a few scenarios. A first scenario is where the fiber is scanned through a large sample volume, while measuring cell fluorescence. Cells with a given signature (fluorescence), for instance from circulating tumor cells, can then be collected. Another scenario of interest is one where the system is scanned through a distribution of cells, to monitor a cell-concentration increase or decrease over time, as an effect of a treatment. A third case could be an assay with hundreds of wells to be analyzed and cells selectively picked from the wells. Finally, we have a scenario closer to an in-vivo, clinical diagnostic situation, where tumor cells are identified as part of solid tissue^28,29^. The work described here is a step towards these possible applications.

Experimentally, our new search-and-pick device was evaluated by selective collection of fluorescently labeled cancer cells from a suspension of unlabeled cells in a fully automated manner. Furthermore, the recovered cells were viable and readily available for downstream analysis. The system shows promise for future integration with, for example, previously developed cytometer-based LiF platforms^30,31^ or other molecular point-of-care diagnostic methods. We expect this system to be used in the future to collect patient samples for in-vitro diagnostics from remote and sensitive regions where biopsy is harder to perform. Combining this LiF tool with other technologies would pave the way for more personalized therapies.

## Materials and methods

### Design and fabrication of the fiber probe

**Fig. 1:**
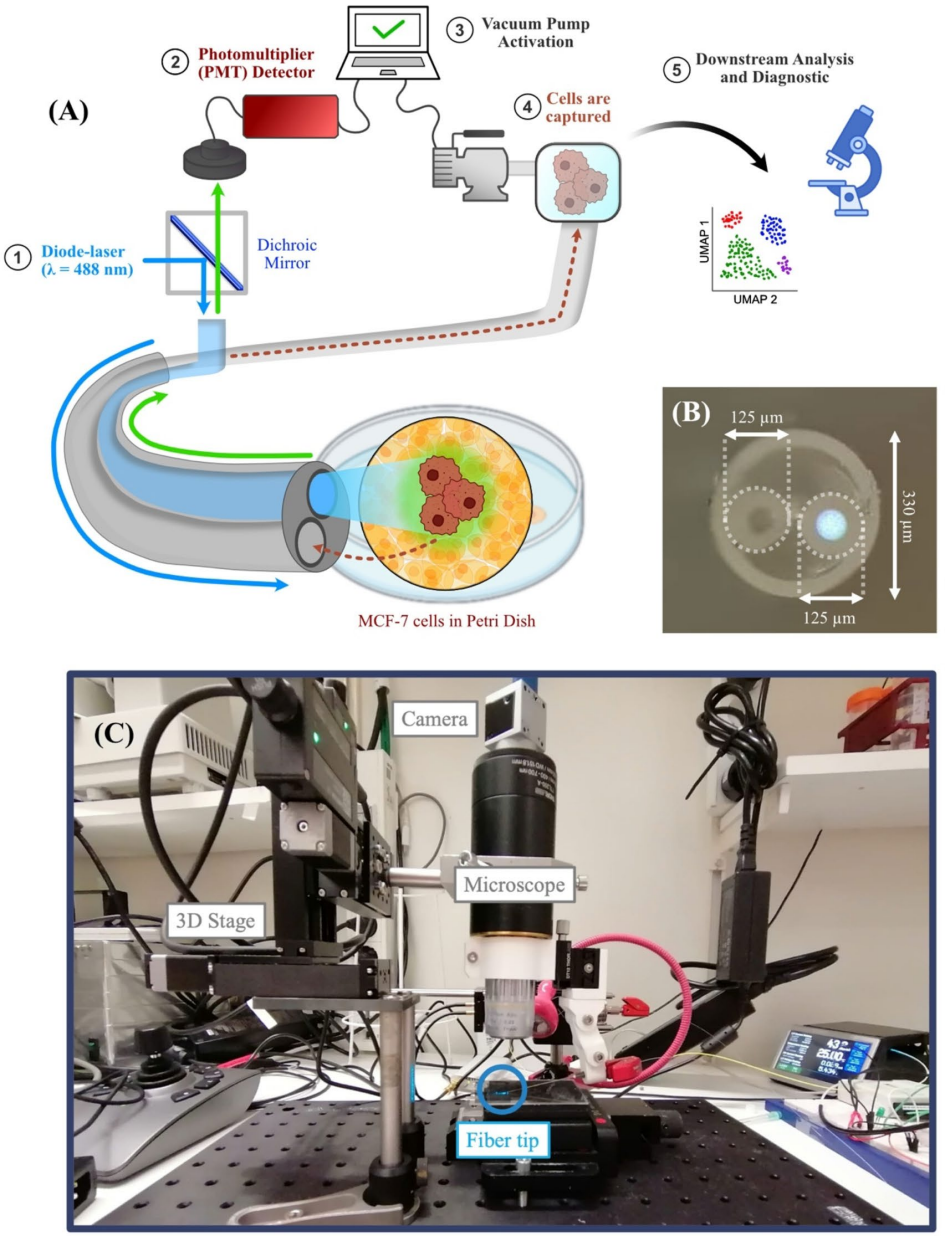
(**A**) Schematic of the LiF system. (1) Light from a 488 nm fiber-coupled diode laser is coupled to the MMF and illuminates a solution with cells. (2) Fluorescence from labeled cells is collected backwards through the same fiber and guided to the PMT detector. (3) When the fluorescent signal is above a set threshold, the vacuum pump is activated, and negative pressure is applied to the capillary. (4) Cells are then captured by the capillary and transported to a reservoir. (5) Upon collection, the cells can be used for further downstream analysis and diagnosis. (**B**) Microscope image of the cross-section of the assembled fiber probe. The capillary is seen to the left and the excitation fiber to the right with the core illuminated by the laser. (**C**) Picture of the LiF platform.

The LiF device introduced in this work is illustrated in Fig. 1A. It consists of an optical fiber probe made of two fibers: (i) a multimode silica fiber (MMF) with a core and cladding diameter of 62.5 μm and 125 μm, respectively, and (ii) a silica fiber capillary with an inner and outer diameter of 90 μm and 125 μm, respectively. The two fibers were inserted side-by-side in a housing capillary with an inner diameter of 255 μm and an outer diameter of 330 μm. The flat-cleaved fibers were secured inside the housing capillary using a UV-cured glue and then treated with hydrophobic liquid (Sigmacote^®^; Sigma-Aldrich) to prevent clogging by aggregation of cells to the capillary. A MMF coupled blue laser (488 nm) was used to illuminate the sample and collect the green fluorescence light (~ 517 nm) from fluorescently labeled cells. The fiber capillary was connected to a vacuum pump and operated in a similar fashion as described in refs^26,27^. The pump activation system is similar to that described in Ref^27^. A solenoid valve is connected between the pump and the fiber capillary. The pump is running continuously, and the solenoid valve is switched on or off depending on when we want to suck the cell into the capillary. The switch is controlled using the PC through a Python program and activated when the detected signal goes above threshold. The inner diameter of the capillary was chosen to be 90 μm to allow for the capture of single cells and smaller cell clusters while limiting the volume of liquid collected and minimizing pressure-drop and clogging problems. A cross-section image of the fully assembled fiber probe can be seen in Fig. 1B. The details of the components used in the setup and the part numbers of the devices used are presented in Table S1. The simplicity of our design allows for low-cost fabrication using standard fibers with no need for clean-room facilities^30^. It also allows for an easy addition of more components with different functionalities in the future.

### Cell culture

For conducting the experiments described below, MCF-7 cells were acquired from the American Type Culture Collection (ATTC) and cultured in Dulbecco’s modified Eagle’s medium-F12 (DMEM; Sigma-Aldrich) supplemented with 10% Fetal Bovine Serum (FBS; Sigma-Aldrich) and 1% penicillin-streptomycin 100 U/mL (Sigma-Aldrich), in a humidified incubator with 5% CO_2_, at 37^o^C. Once confluency of 70–80% was reached, the cells were incubated with a solution of Calcein-AM (2.5 µM; Sigma-Aldrich) for 20 min, subsequently dissociated and collected from the cell culture flask using a 0.25% solution of Trypsin-EDTA (Sigma-Aldrich) and separated by centrifugation at 1300 rpm (approximately 272 g) for 5 min. The cells were then resuspended in either DMEM or a Dulbecco’s phosphate-buffered saline (PBS; Sigma-Aldrich) solution and diluted to the desired concentration. Cell and particle concentrations were determined using Trypan Blue Stain (0.4%) with the Countess 3 automated cell counter (ThermoFisher Scientific), and the viability of cells captured using the fiber probe was assessed through an exclusion test on glass slides using the Trypan Blue Stain. Calcein-AM was the fluorophore for testing since it is commonly used for routine cell staining, especially for assessing cell viability^32–34^, despite the fluorescence signal decay under prolonged exposure. To further assess if captured cells could grow when cultured, a 10 µL sample was taken from a solution of approximately 10^6^ cells/mL using the fiber probe, followed by 4 days of culture under the aforementioned conditions, at a starting culture concentration of approximately 2000 cells/mL.

### Experimental setup, procedure, and validation

During the experiments the LiF fiber probe was mounted on a motorized 3D-translation stage (Zaber XYZ) controlled by a joystick, allowing it to be swept through the sample with micrometer precision. While scanning the solution, the fluorescent signal was continuously collected through the MMF, filtered by a dichroic mirror, and detected using a photomultiplier tube (PMT). When the detected signal exceeded a predetermined threshold, it indicated that a fluorescent particle was excited in front of the fiber tip. The vacuum pump was subsequently activated, thus applying a negative pressure (~ 350 mbar) to the capillary inducing inward flow of the solution into the capillary. The collected sample volume depended on the pump activation time. a A microscope equipped with a CCD camera was mounted above the tip of the fiber probe to monitor the experiment. Detailed information on the components and devices used is presented in Table S1.

The volume captured as a function of pump activation time was experimentally verified by measuring the distance traveled by a colored dye upon manual activation of the pump and calculating the corresponding volume. The pressure applied by the pump was ascertained using a microfluidic in-line pressure sensor (Fluigent).

Initial measurements were carried out to validate the proper functioning of the system. Green-fluorescent polystyrene particles (10 μm diameter Fluoro-Max Microspheres, Thermo Scientific) were used in a PBS solution and afterward in a DMEM solution. Similarly, tests were carried out with Calcein-AM labeled MCF-7 cells at a concentration of 2.38 × 10^6^ particles/mL in PBS and then in a DMEM solution containing phenol-red for cell growth. The specificity of the system was tested by sweeping the fiber component through a solution of unlabeled cells (concentration ≈ 2.42 × 10^6^ cells/µL) spiked with resuspended Calcein-AM labeled cells (concentration ≈ 1.08 × 10^5^ cells/µL) in a small petri dish.

We remark that if the system were to measure the sum of the fluorescence signals from many cells to reach a threshold, then there could be a limit-of-detection problem for low concentrations. In the present case, however, the system reads a single cell at a time, and the threshold is reached when the distance between the cell and the fiber tip is sufficiently short. Therefore, the limit of detection is a single cell, care is taken to ensure that the SNR of the measurement of a labelled cell very near the fiber tip is ~ 3, high enough to define a threshold to enable selective collection.

## Results

### Optical characterization

**Fig. 2:**
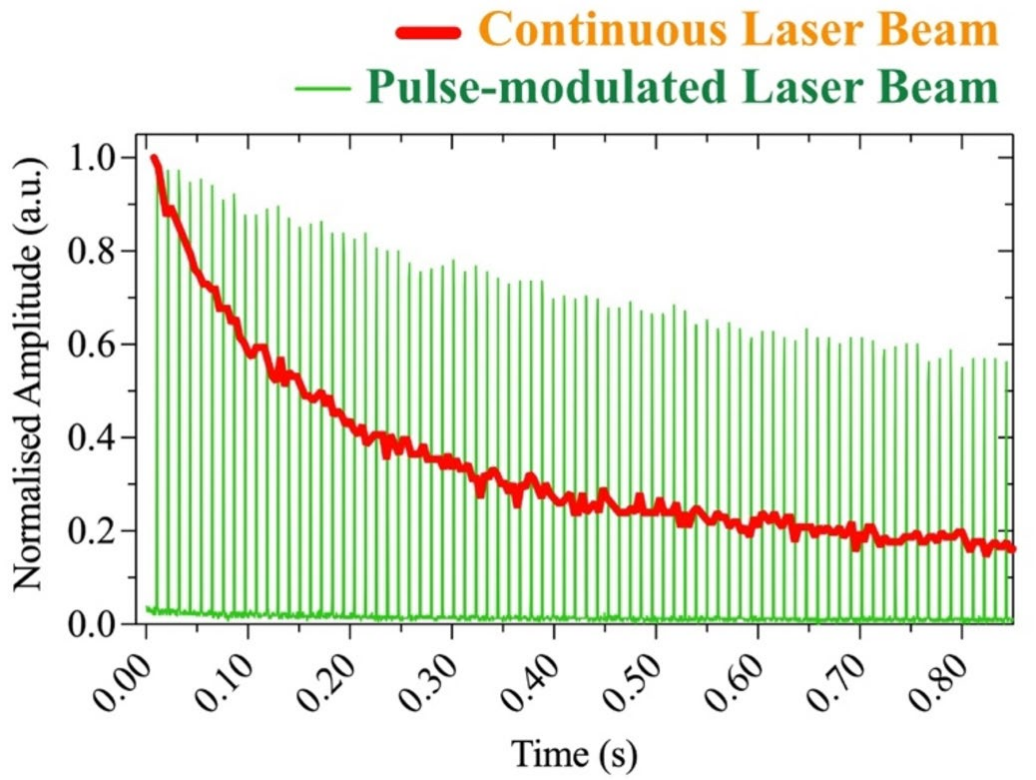
Detected signal from labeled cells vs. excitation time using continuous illumination (red), or an On-Off modulated laser beam (10% duty cycle) with a frequency of 100 Hz (green).

The initial fluorescence measurements performed with fluorescent particles and Calcein-AM labeled cells were compared to the background signal (Fig. S1). Note that the fluorescence lifetime of most dyes is only a few nanoseconds, but under constant pumping, the fluorescently labeled polystyrene particles showed a relatively stable signal over time, with an initial amplitude of ~ 175 mV. In contrast, continuous illumination of the cells led to bleaching, as can be seen in Fig. 2. Calcein-AM labeled cell measurements performed in PBS and DMEM with phenol-red solutions showed comparable amplitudes and a similar trend of decaying fluorescence with exposure time. Starting at ~ 100 mV, the fluorescence decayed to the calculated limit-of-detection (LoD) of approximately 20 mV after two minutes of exposure. The well-known photobleaching of dyes exposed to continuous pump light is a shortcoming of many experiments^35,36^. Here it limits the time available for fine tuning the fiber tip position in search of the optimal collection point. Attempts to substitute dyes by light-emitting moieties, such as quantum dots, lanthanides, and rare earths that exhibit very slow photodegradation have been made^37–39^, but biocompatibility is still an unsolved problem. Instead, to obtain longer measurement time and reduce photobleaching we made use of pulsed excitation, as discussed below.

The fluorescence decay is dose-dependent^32^ and under continuous exposure the fluorescence signal from the cells halved in ~ 130 ms, as seen in Fig. 2, in red. To enable longer measurement time, we illuminated the fluorescent dye for 1-ms at a rate of 100 Hz, thereby decreasing the overall exposure time ten-fold in comparison to continuous pumping. The decay of the fluorescence envelope is then correspondingly slower, with a half-time decay of 1.06 s, see the pulsed signal in Fig. 2, in green. This pulsed excitation procedure is quite general, and it was valuable to allow searching for the optimum point for cell collection, as discussed below. It could also help prolonging the dye usage in other applications, such as in microscopy.

### Fluidic setup characterization

**Fig. 3:**
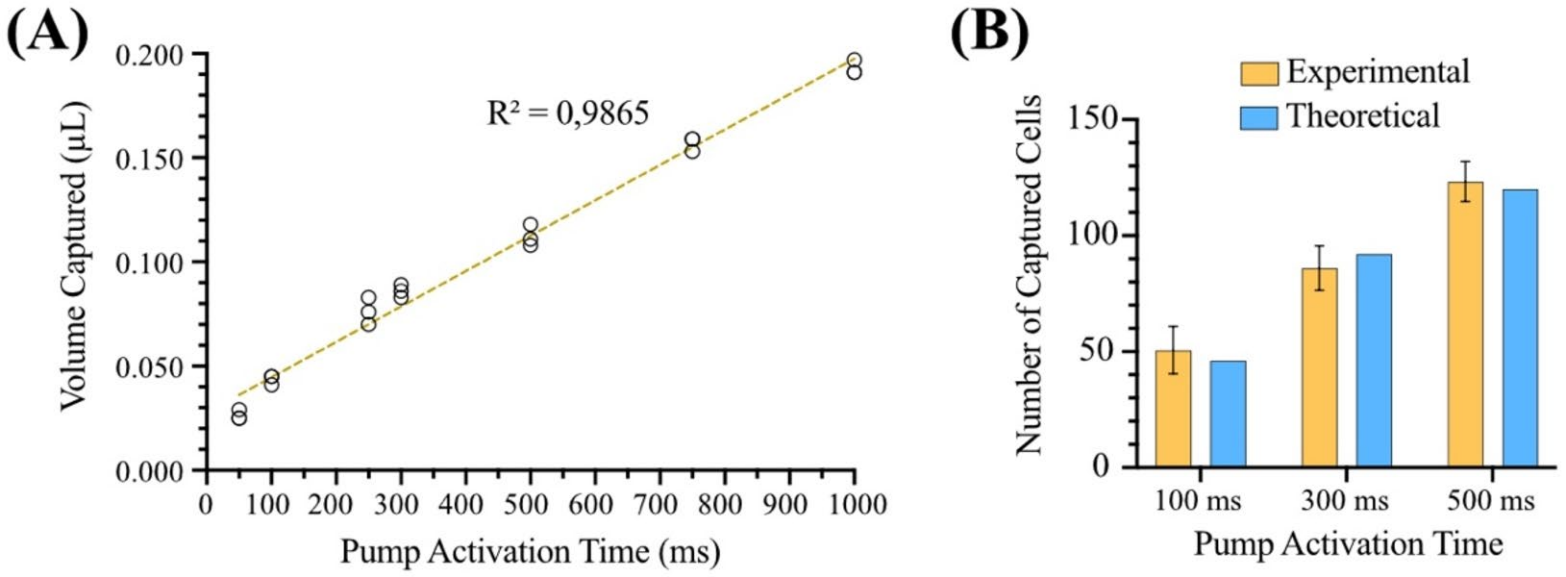
(**A**) Volume captured in the capillary for different pump activation times. Each circle represents one individual measurement. (**B**) Total number of captured cells for each pump activation time compared to the expected number, assuming the capture volumes experimentally determined in (**A**) for a concentration of 1.07 × 10^6^ cells/mL. The cells were counted manually using Trypan Blue Stain.

A pump was used to apply differential negative pressure to the capillary to capture the fluorescent particle of interest. A correlation between pump activation time and the volume captured in the capillary was experimentally established, as shown in Fig. 3A. A minimum volume of 26 nL was collected using a pump activation time of 50 ms. Experiments capturing fluorescent cell were performed subsequently, for three different pump activation times in a continuously stirred solution containing 1.07 × 10^6^ cells/mL, while the LiF fiber probe was kept stationary. The number of captured cells was determined through a trypan-blue stain counting procedure and compared to the theoretically expected number. The results are shown in Fig. 3B. Except for a dead volume that affects the results for short activation periods, the collection grows linearly in time and so does the number of retrieved cells (within ~ 10%).

### Detection of labeled cells

**Fig. 4:**
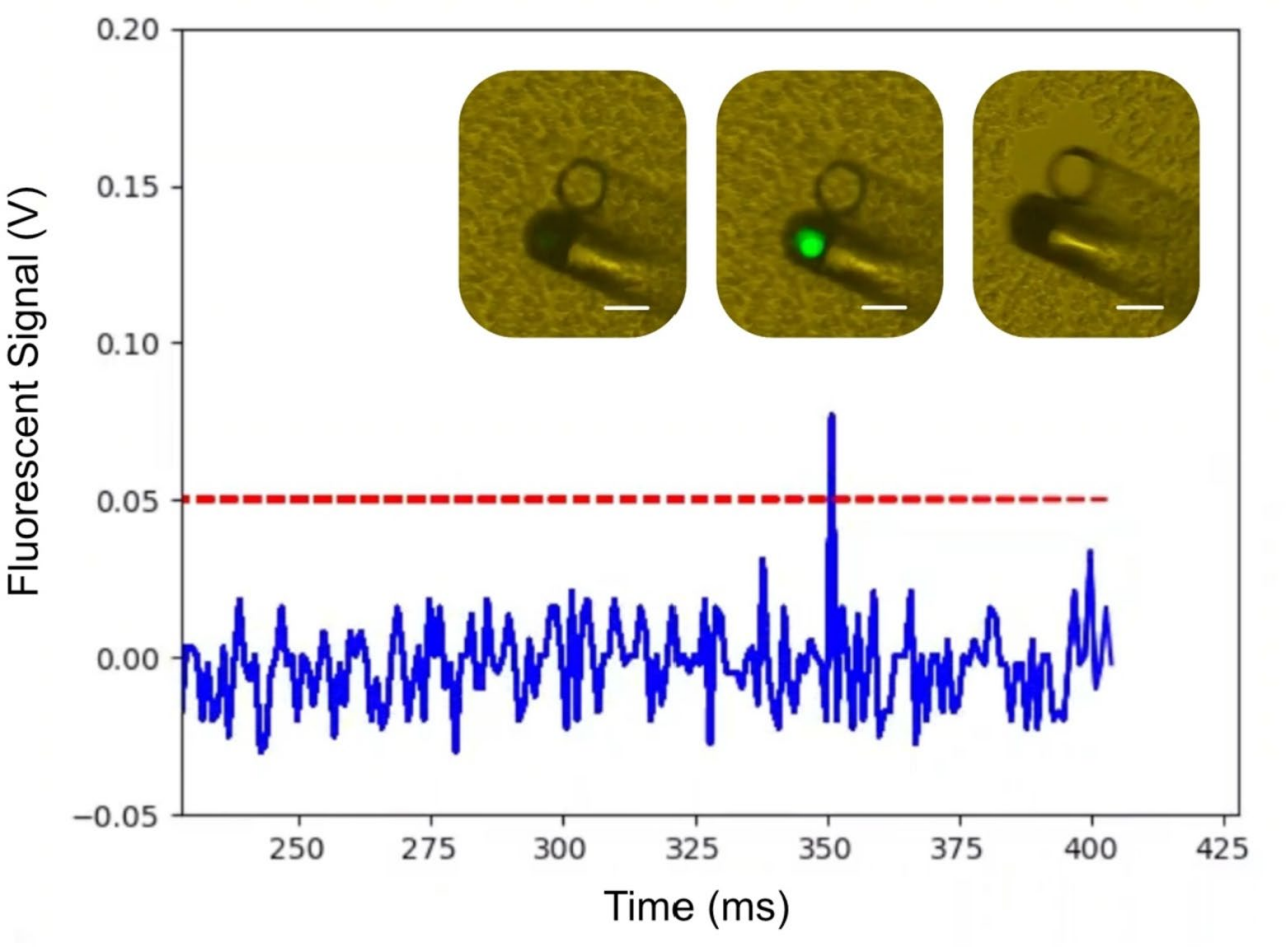
Fluorescence signal vs. time when the fiber is scanned through the solution with labeled and unlabeled cells. The blue line shows the signal collected through the MMF. The red dashed line marks the threshold for activation of the vacuum pump. At instant 350 ms, a cell is detected and collected. The three microscopy images represent corresponding images; prior to detection (left); when a cell is excited (middle); and after collection (right). The scale bar is 100 μm for all images.

After characterization the system was used to screen a solution of unlabeled cells spiked with a small amount of fluorescently labeled cells in a ratio of 20:1. The fiber probe was scanned through the solution using a joystick connected to the 3D stage. A microscope was used to image the fiber tip as it was scanned through the sample. A video of the fiber probe picking up a fluorescently labeled cell from this solution is available in the supplementary data (Supplementary Video 1). Selected images of each of the important events are presented in Fig. 4. First, we see the fiber probe approaching a fluorescent cell, and at time = 350 ms the system detects its presence and activates the vacuum pump. The targeted cell is then captured.

### Viability of captured cells

**Fig. 5:**
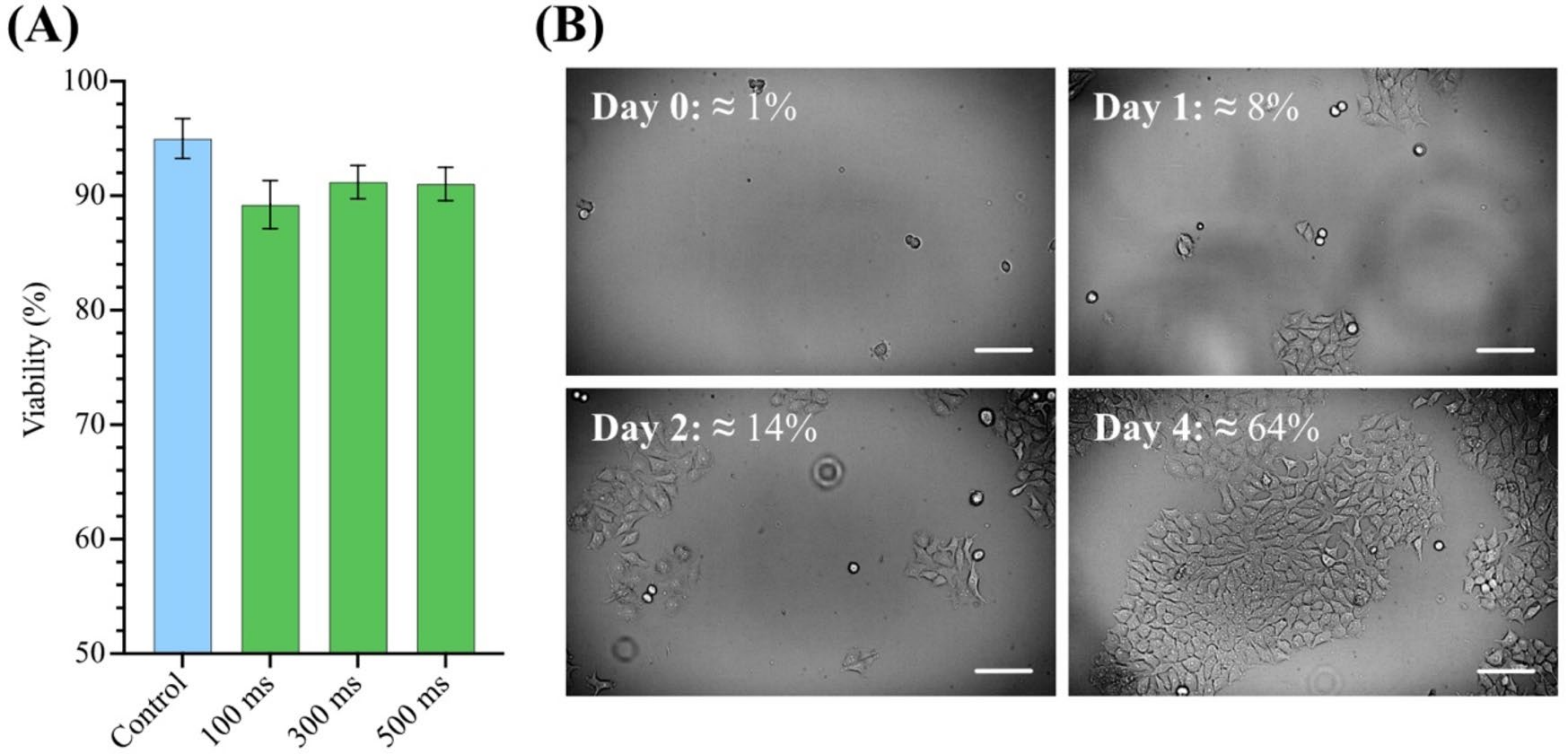
(**A**) Viability of MCF-7 cells captured using three different pump activation times. The control solution (blue) consisted of cells kept at room temperature, but not captured using our LiF system. Viability was determined by manual counting using a standard Trypan Blue live-dead assay. Error bars correspond to the standard deviation of three consecutive measurements. (**B**) Captured MCF-7 cells grown over four days. Each day corresponds to an approximate 24-hour period after the image from Day 0 was taken. Confluency of the cells is presented for each day of growth. Scale bars correspond to 100 μm. Images were acquired using a ZOE Fluorescent Cell Imager (Bio-Rad).

The primary objective of cell collection is to enable downstream analysis. For cancer the quality of cells is particularly important for an accurate diagnosis. For that reason, we determined the viability of the captured cells. An exclusion test using the Trypan Blue Stain on glass slides was done by manually counting the cells alive or dead for three samples collected using three different pump activation times (100 ms, 300 ms and 500 ms; Fig. 5A). The results were also compared to a control cell solution that was kept at room temperature, but not subjected to any capture by the system. The growth of cells after capture was also tested. Approximately 10 µL were captured from a solution containing 10^6^ cells/mL. These cells were then cultured for 4 days at a starting concentration of 2000 cells/mL, see Fig. 5B. The results show that a vast majority of cells remain viable after collection through the LiF system and can grow for more than 4 days when cultured.

## Discussion

### LiF setup characterization

The pulse excitation technique described above can help the analysis of various fluorescent samples hindered by photobleaching and low signal-to-noise ratio (SNR). It should be mentioned that the frequency (100 Hz) and pulse duration (1 ms) used in the present work were chosen for ease of implementation. The response times of the pump laser, photodetector, and oscilloscope used in our experiments were all below 50 ns. Therefore, to minimize photobleaching further, pumping of the dye can be done with shorter pulses and at higher frequency. One could also reduce the pulse repetition frequency while increasing the peak power of the pump laser by the same factor. This would increase the SNR while keeping the exposure constant. Furthermore, a sample-and-hold circuit could be used with the detection system, to render the trace continuous that is pulsed on and off here. Nonetheless, our pulsing technique allowed measurements longer than 5 min (Fig. S2), making it more compatible with longer diagnostic procedures.

The specificity of the fluorescence-based triggering of the suction pump benefits from the fact that cells far from the tip generate a signal that is too weak to exceed the threshold for collection. If the fiber core and the fluorescent cell are treated as point sources, the collected signal drops with the fourth power of the distance^27^. A modest increase of the cell distance by 3 times leads to a two orders-of-magnitude reduction in the detected fluorescence, and the threshold for cell suction is not reached. This principle is then conveniently explored in our set-up, since only cells near the collection point are suck into the capillary, minimizing the accompanying volume of liquid that inevitably is collected with the cell and improving the specificity of the system. The finite core size of the MMF and the distance between the centers of the core and of the capillary alter this dependence for short distances. A complete system design could take this into account when setting the threshold level for pump activation.

The minimum volume collected that guarantees that the cell is captured when the pump activates is approximately half a sphere (2/3 π*r*^3^) of radius *r* equal to the distance between the cell and the capillary tip. This assumes that the flow into the capillary is uniform from all directions in front of the capillary tip and zero from behind the tip. This expression allows estimating the volume collected together with the cell that triggers the system. For example, for a volume collected of 26 nL (smallest value in Fig. 3A), *r ~* 232 μm. If the concentration is known, the minimum number of cells that accompany the cell of interest can be evaluated.

### Detection, capture, and viability of cells

As shown in Media 1 and Fig. 4, the system can identify a fluorescently labeled cell in an unlabeled matrix, trigger the suction pump, and capture the target cell once the signal is above the predefined threshold. Importantly, the cells proliferate after capture, reaching a confluency of above 70% after 4 days. Furthermore, the captured cells keep a high viability of ~ 90%, enabling possibilities for diagnostic methods that require the culturing of patient samples, such as drug sensitivity tests or functional assays^40,41^.

Further development of this LiF platform as a diagnostic tool could focus on label-free cell identification, analyzing the collected back-scattered signal with the help of AI^15^ and on how to improve the cytologic sampling, since only collection in liquid samples was demonstrated to date. The latter could make use of laser-produced micro incisions^42–44^, or the use of a micro brush^45,46^ to release cells from tissue.

## Conclusion

In this work, we introduced a LiF device where the combination of a light-guiding fiber and a fluid-handling capillary enables the detection and capture of fluorescently labeled cells while preserving their viability. By using pulsed excitation, the measurement time is sufficiently increased to search and pick labeled cells in a mixture with unlabeled cells. Because of its small dimensions, the device can potentially be used in remote and sensitive regions where biopsy is difficult. The system shows potential for use in a clinical setting, where small samples of cells can be collected in vivo for further analysis, a step in the direction of a tool for personalized therapy.

## Electronic supplementary material

Below is the link to the electronic supplementary material.

Supplementary Material 1 (Media 1)

Supplementary Material 2

## Data Availability

Data sets generated during the current study are available from the corresponding author on reasonable request.
