# Supplementary material for: Lab-in-a-Fiber detection and capture of cells
Source: Sci Rep. 2025 Mar 20;15:9694. doi: 10.1038/s41598-025-92585-6 (PMC11926341; doi:10.1038/s41598-025-92585-6)
Supplement: Supplementary file 2 — Supplementary Material 2 [file 41598_2025_92585_MOESM2_ESM.docx]

Supporting Information

**LAB-IN-A-FIBER DETECTION AND
CAPTURE OF CELLS**

João C. Varela^1, 2†*^, Achar V. Harish^3, 4†^, Pawel Maniewski^3^, Timothy Gibbon^4^, Oana Tudoran^1,5^, Rainer Heuchel^6^, Matthias Löhr^6^, Walter Margulis^3, 7^, Aman Russom^1, 2^, Fredrik Laurell^3^

^1^Division of Nanobiotechnology, Department of Protein Science, Science for Life Laboratory, KTH Royal Institute of Technology, Solna, Sweden

^2^AIMES Center for the Advancement of Integrated Medical and Engineering Sciences at Karolinska Institutet and KTH Royal Institute of Technology, Stockholm, Sweden

^3^Department of Applied Physics, KTH Royal Institute of Technology, Stockholm, Sweden

^4^Research Institutes of Sweden (RISE), Stockholm, Sweden

^5^Department of Genetics, Genomics and Experimental Pathology, The Oncology Institute “Prof. Dr. Ion Chiricuta”, Cluj-Napoca, Romania

^6^Department of Cancer Medicine, Division for Upper GI, Karolinska University Hospital, Stockholm, Sweden;

^7^Catholic University of Rio de Janeiro PUC-Rio, Rio de Janeiro, Brazil

^†^These authors contributed equally to this work; ^*^E-mail: joao.varela@scilifelab.se

**Table S1:** Components used to assemble the Lab-in-a-Fiber setup used for the detection and capture of cells.

**
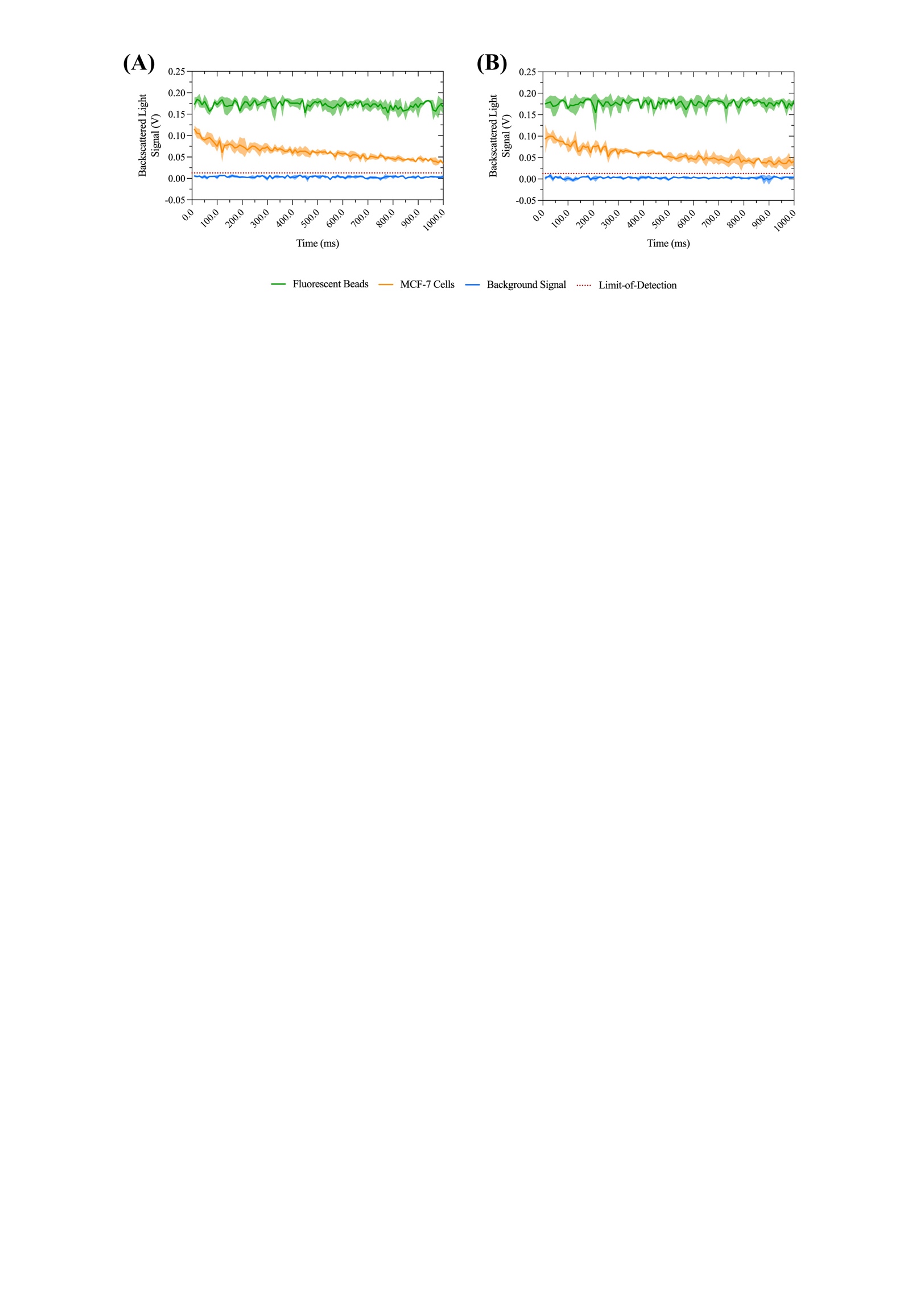
Figure S1:** Fluorescent signal detected by the PMT detector (V) vs. time in a solution of **(A)** PBS buffer, and **(B)** DMEM cell culture media. The plots are average of 10 datapoints, each corresponding to 1 ms. The shaded areas correspond to the standard deviation (n = 3). Limit-of-Detection corresponds to the average value of the background signal plus three times the average standard deviation.

**
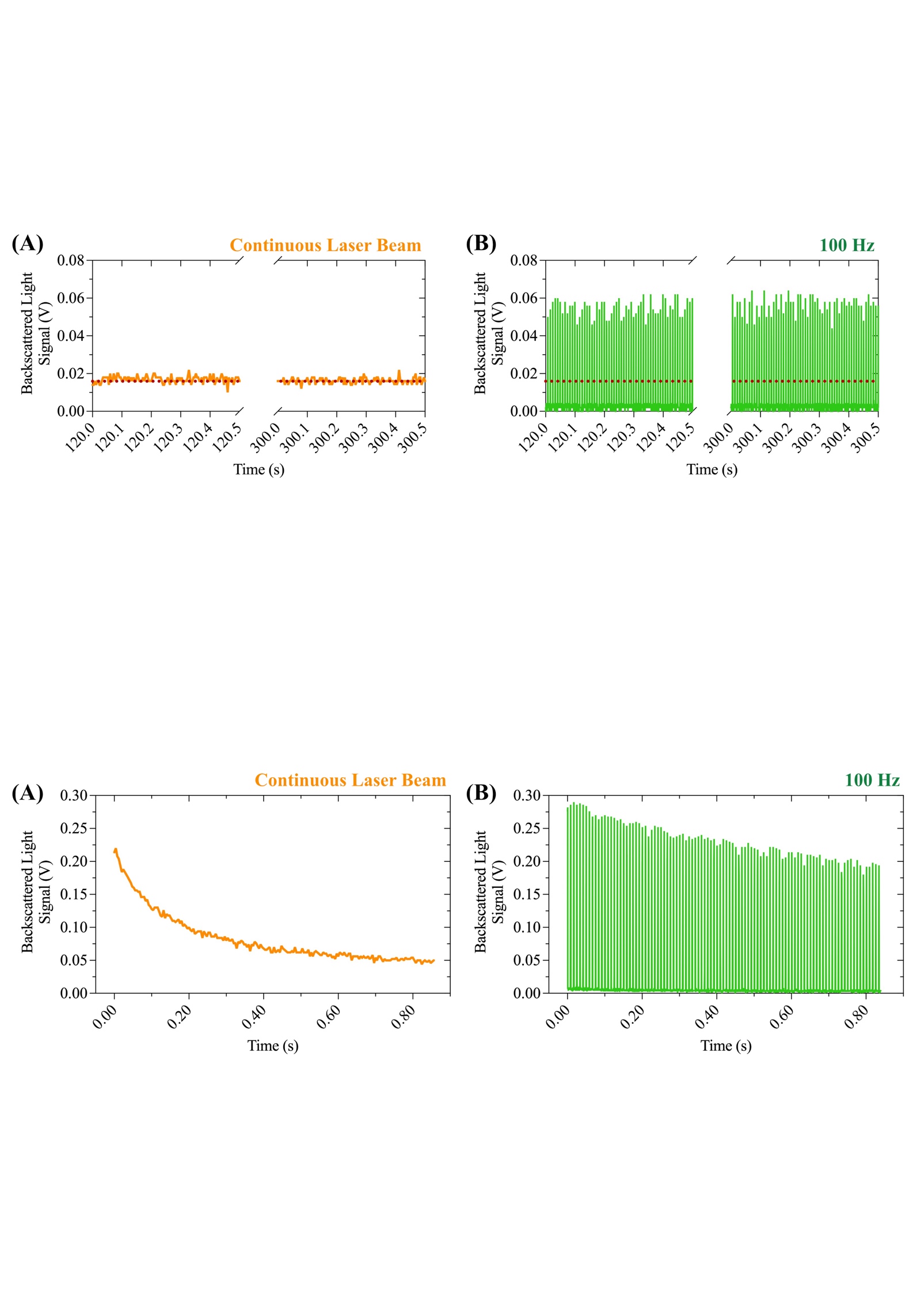
**

**Figure S2:** Detector signal from the PMT detector (V) after a period of two and five minutes using continuous exposure **(A),** or on-off modulated excitation (10% duty cycle) with a frequency of 100 Hz **(B)**. Limit-of-Detection is the red-dotted line. It corresponds to the average value of the background signal plus three times the average standard deviation.

**Figure S3:** Excitation (dotted line) and emission (continuous line) spectrum of the used Calcein-AM (Sigma-Aldrich), adapted from the information provided by the manufacturer.
